# To the non-local theory of cold nuclear fusion

**DOI:** 10.1098/rsos.140015

**Published:** 2014-10-15

**Authors:** Boris V. Alexeev

**Affiliations:** Department of Physics, Moscow Lomonosov University of Fine Chemical Technologies, prospect Vernadskogo 86, Moscow 119571, Russia

**Keywords:** foundations of the theory of transport processes, generalized hydrodynamic equations, basements of non-local physics, cold nuclear fusion, solitons in the sound waves

## Abstract

In this paper, we revisit the cold fusion (CF) phenomenon using the generalized Bolzmann kinetics theory which can represent the non-local physics of this CF phenomenon. This approach can identify the conditions when the CF can take place as the soliton creation under the influence of the intensive sound waves. The vast mathematical modelling leads to affirmation that all parts of soliton move with the same velocity and with the small internal change of the pressure. The zone of the high density is shaped on the soliton's front. It means that the regime of the ‘acoustic CF’ could be realized from the position of the non-local hydrodynamics.

## 2. Introduction

In 1989, two electro-chemists, Martin Fleischmann and Stanley Pons [1] announced about nuclear fusion reactions between deuterium nuclei in a table-top experiment, under ordinary conditions of temperature and pressure, by using electrochemistry. The experimental evidence consisted of the production of large amounts of heat, which could not be attributed to chemical reactions.

The reactions were termed ‘cold fusion’ (CF), by comparison with the high temperature of thermonuclear fusion. The typical chain of nuclear reactions can be written as follows:

$$\begin{array}{rl} & \text{d}+\text{d}\to 3\text{He}+\text{n}+4.0\;\text{MeV},\\ & \text{d}+\text{d}\to \text{t}+\text{p}+3.25\;\text{MeV},\\ & \text{t}+\text{d}\to 4\text{He}+\text{n}+17.6\;\text{MeV}\\ \text{and}\; & 3\text{He}+\text{d}\to 4\text{He}+\text{p}+18.3\;\text{MeV}. \end{array}$$

Obviously, the following criteria need to be met in order to establish conventional thermonuclear deuterium fusion unquestionably:

(1) the experiment has been repeatable by other investigators;
(2) there has to be a significant neutron emission statistically well above background level;
(3) the energy spectrum of the detected neutrons must match the energy spectrum of neutrons produced in deuterium fusion; and
(4) there should be no neutron sources in the laboratory that can confuse the fusion neutron measurements.

Many scientists cannot reproduce the mentioned experimental results. The scientific community concluded that there were no nuclear reactions and that the reported experiments were in error. CF was considered an example of wrong science. This produced a partition between the traditional scientific world and the community which continued the CF research. In the 20 years elapsed since the announcement by Fleischmann and Pons that the excess enthalpy generated in the negatively polarized Pd-D-D_2_O system was attributable to nuclear reactions occurring inside the Pd lattice, there have been reports of other manifestations of nuclear activities in this system. In particular, there have been reports of tritium and helium-4 production; emission of energetic particles, gamma or X-rays and neutrons; as well as the transmutation of elements. Reproducibility was improved and Mosier-Boss *et al*. [2] declared as result of accurate measurements about the real existence of the ‘Fleischmann–Pons effect’.

An other system known as Energy Catalyzer (also called E-Cat) was devised by inventor Andrea Rossi [3] with support from physicist Sergio Focardi. Rossi and Focardi said the device worked by infusing heated hydrogen into nickel powder, transmuting it into copper and producing heat.

Many scientists were convinced of the unlikelihood of a chemical reaction being strong enough to overcome the Coulomb barrier, the lack of gamma rays, the lack of explanation for the origin of the extra energy, the lack of the expected radioactivity after fusing a proton with ^58^Ni and so on. The main secret of the Rossi's device consists in the catalysts used in E-Cat, but the scientific community has no access to information about the catalysts in Rossi's device.

In the following investigation, I intend to construct the non-local theory of CF for the system which can be analysed by the methods of theoretical physics. Then we have no reason to discuss the physical systems with unknown influence of unknown catalysts. Practically, considering above the CF direction of investigation can be called ‘catalysts CF’. Much more interesting for the theoretical investigation is the situation when light nuclei are forced together using the external forces like effects of cavitations, shock waves or combination of the possible force effects. In this case, particles will fuse with a yield of energy because the mass of the combination will be less than the sum of the masses of the individual nuclei.

Many successful cavitation-induced fusion experiments have been performed and reported in peer-reviewed literature (e.g. [4–14]). Russian publications were practically never translated into English and therefore Russian-Soviet work is not widely known or cited in the West.

Subsequent work was carried out by Bityurin *et al*. [4] at the Joint Institute for High Temperatures of the Russian Academy of Sciences. The group studied the effect of shockwaves on deuterated liquid (D_2_O) with high (20–95%) bubble content. Their experimental set-up includes admission of deuterium bubbles into deuterated liquid and crushing them with a shockwave generated via an explosion of a semicircular wire due to high current pulse. The resulting shockwave propagates in the bubble–liquid phase and focuses much more strongly than in the pure liquid due to shockwave amplification effects in the gaseous phase. As is well known, the fusion of D atoms results in the emission of a proton, helium-3, a neutron (of 2.45 MeV energy) and tritium. Protons (in the MeV range) are charged particles which cannot traverse more than 1 mm in the liquid before getting absorbed and, therefore, cannot be measured with detectors outside of the apparatus. The same problem holds true for helium-3 atoms which are non-radioactive and difficult to detect in small quantities. Neutrons are uncharged particles which can leak out of the test chamber and can be detected with suitable instrumentation. Also, tritium being a radioactive gas which remains in the test liquid can be counted for *β*-decay activity (if a suitable state-of-the-art beta spectrometer is available). Therefore, testing was initiated systematically for monitoring the key signatures consisting of tritium and neutron emissions. The group used Indium (*β*-decay) detectors to measure neutron flux and estimate total neutron yield at 10^8^–10^10^ per explosion.

Naturally, the possibility of attaining nano-scale nuclear fusion in the cores of collapsing bubbles in liquids leads to tremendous difficulties for the theoretical description. Really, we should apply the unified theory that realizes the ‘through’ description from macroscopic level to the nuclei scale. This description cannot be realized not only on the ‘classical’ hydrodynamic level but on the level of Schrödinger quantum mechanics (SQM) because SQM is not applicable to the nuclei problems. Delivery of the main principles of the unified non-local theory in the concentrated form in my plenary lecture can be found [15].

From this point of view, there is no reason to estimate fusion efficiency by solving the Rayleigh–Plesset–Keller (RPK) differential equation for bubble collapse. By the way, the RPK equation must be solved numerically together with the equation of state for the bubble gas. No surprise that the resulting solution is quite sensitive to the choice of the equation of state during the last stage of collapse. But the equation of state for ideal gas cannot be used for this stage which is the most interesting stage from the standpoint of nuclear fusion.

The adversaries of the cavitation-induced fusion affirm that theoretical physics does not lead to the acoustic regimes providing CF and all the effect is within the experimental error.

The main objective is to investigate the dynamics of matter under the influence of the sound wave by the methods of non-local physics.

## 3. Investigation of the soliton movement under action of the sound wave

Let be the plane sound wave interact with matter. In this case, we can say about the sound pressure *P* which can be observed for example in the stiff tube. This pressure *P* was calculated by Rayleigh [16–18] and can be written as

3.1 $$P=\frac{\gamma +1}{8}\rho_{m}v^{2}=(\gamma +1)E_{k},$$

where *ρ*_*m*_ is the density of a surrounding medium without perturbations, *v* is amplitude of the sound particle velocity in the wave antinodes, *E*_*k*_ is the time- and space-average of the kinetic energy density of the sound wave, *γ*=*c*_*p*_/*c*_*V*_ (the ratio of the specific heat at constant pressure to the specific heat at constant volume).

Non-local hydrodynamic equations have the form [15,19,20]:

(continuity equation)

3.2 $$\begin{array}{rl} & \frac{\partial }{\partial t}\left\{\rho -\tau \left[\frac{\partial \rho }{\partial t}+\frac{\partial }{\partial \text{r}}\cdot (\rho {\text{v}}_{0})\right]\right\}+\frac{\partial }{\partial \text{r}}\cdot \left\{\rho {\text{v}}_{0}-\tau \left[\frac{\partial }{\partial t}(\rho {\text{v}}_{0})+\frac{\partial }{\partial \text{r}}\cdot \rho {\text{v}}_{0}{\text{v}}_{0}+\overset{↔}{I}\cdot \frac{\partial p}{\partial \text{r}}-\text{F}\right]\right\}=0, \end{array}$$

(motion equation)

3.3 ∂∂t{ρv0−τ[∂∂t(ρv0)+∂∂r⋅ρv0v0+∂p∂r−F]}−Fρ[ρ−τ(∂ρ∂t+∂∂r⋅(ρv0))]+∂∂r⋅{∂∂tρv0v0+pI↔−τ[∂∂t(ρv0v0+pI↔)+∂∂r⋅ρ(v0v0)v0+2I↔[∂∂r⋅(pv0)]+∂∂r⋅(I↔⁡pv0)−Fv0−v0F]}=0,

(energy equation)

3.4 $$\begin{array}{rl} & \frac{\partial }{\partial t}\left\{\rho v_{0}^{2}+3p-\tau \left[\frac{\partial }{\partial t}(\rho v_{0}^{2}+3p)+\frac{\partial }{\partial \text{r}}\cdot (\rho v_{0}^{2}{\text{v}}_{0}+5p{\text{v}}_{0})-2\text{F}\cdot {\text{v}}_{0}\right]\right\}\\ & \;+\frac{\partial }{\partial \text{r}}\cdot \{\rho v_{0}^{2}{\text{v}}_{0}+5p{\text{v}}_{0}-\tau [\frac{\partial }{\partial t}(\rho v_{0}^{2}{\text{v}}_{0}+5p{\text{v}}_{0})+\frac{\partial }{\partial \text{r}}\cdot \left(\rho v_{0}^{2}{\text{v}}_{0}{\text{v}}_{0}+7p{\text{v}}_{0}{\text{v}}_{0}+pv_{0}^{2}\overset{↔}{I}+5\frac{p^{2}}{\rho }\overset{↔}{I}\right)\\ & \;-2\text{F}\cdot {\text{v}}_{0}{\text{v}}_{0}-2\frac{p}{\rho }\text{F}\cdot \overset{↔}{I}-v_{0}^{2}\text{F}-3\frac{p}{\rho }\text{F}]\}-2\left\{\text{F}\cdot {\text{v}}_{0}-\tau \left[\frac{\text{F}}{\rho }\cdot \left(\frac{\partial }{\partial t}(\rho {\text{v}}_{0})+\frac{\partial }{\partial \text{r}}\cdot \rho {\text{v}}_{0}{\text{v}}_{0}+\frac{\partial }{\partial \text{r}}\cdot p\overset{↔}{I}-\text{F}\right)\right]\right\}=0, \end{array}$$

where **v**_0_ is the hydrodynamic velocity in the coordinate system at rest, *ρ* the density, *p* the pressure, $\overset{↔}{I}$ unit tensore, **F** the force acting on the unit of volume and *τ* the non-local parameter. Several significant remarks follow.

(1) Equations (3.2)–(3.4) should be considered as local approximation of non-local equations (NLE) written in the hydrodynamic form. NLE include quantum hydrodynamics of Schrödinger—Madelung as a deep particular case [15] and can be applied in the frame of the unified theory from the atom scale to the Universe evolution.
(2) As it follows from relation (3.1), the force **F** (connected with the Rayleigh sound radiation pressure) can be considered as the constant value. Of course it is not a principal restriction. Other approximations can be used including the possibility of the self-consistent solution.
(3) At this stage of investigation, we should answer the question of the principal significance—is it possible to obtain the soliton type of solution with the density growth or not? Then there is no reason to now introduce the consideration of the multi component physical system.
(4) The choice of the non-local parameter needs special consideration [19–22]. The system of equations (3.2)–(3.4) convert in the system of quantum hydrodynamic equations by the suitable choice of the non-local parameter *τ*. The relation between *τ* and kinetic energy [21,22] is used in quantum hydrodynamics:
  3.5 $$\tau =\frac{H}{mu^{2}},$$
  where *u* is the particle velocity and *H* is the coefficient of proportionality which reflects the state of the physical system. In the simplest case, *H* is equal to the Plank constant ℏ and the corresponding relation (3.5) correlates with the Heisenberg inequality. From first glance, the approximation (3.5) is distinguished radically from the kinetic relation known from the theory of the rarefied gases (3.6):
  3.6 $$\tau =\Pi \frac{\upsilon \rho }{p}$$
  which is used for the calculation of the non-local parameter in the macroscopic hydrodynamic case (*υ* is the kinematic viscosity). But it is not a case. In quantum approximation, the value $\upsilon^{\mathrm{qu}}=\hbar /m$ has the dimension (cm^2^ s^−1^) and can be called as quantum viscosity, for the electron species $\upsilon^{\mathrm{qu}}=\hbar /m_{e}=1.1577$ cm^2^ s^−1^. If we take into account that the value $p/\rho ∼\bar{V^{2}}$, then the interrelation of (3.5) and (3.6) becomes obvious.

Let us consider now the one-dimensional non-stationary matter movement under action of the wavefront. In this case, equations (3.2)–(3.5) take the form:

(continuity equation)

3.7 $$\begin{array}{r} \frac{\partial }{\partial t}\left\{\rho -\tau \left[\frac{\partial \rho }{\partial t}+\frac{\partial }{\partial x}(\rho u)\right]\right\}+\frac{\partial }{\partial x}\{\rho u-\tau [\frac{\partial }{\partial t}(\rho u)+\frac{\partial }{\partial x}(\rho u^{2})+\frac{\partial p}{\partial x}-F]\}=0, \end{array}$$

(motion equation)

3.8 $$\begin{array}{rl} & \frac{\partial }{\partial t}\left\{\rho u-\tau \left[\frac{\partial }{\partial t}(\rho u)+\frac{\partial }{\partial x}(p+\rho u^{2})-F\right]\right\}-F+\frac{F}{\rho }\tau \left(\frac{\partial \rho }{\partial t}+\frac{\partial }{\partial x}(\rho u)\right)\\ & \;+\frac{\partial }{\partial x}\left\{\rho u^{2}+p-\tau \left[\frac{\partial }{\partial t}(\rho u^{2}+p)+\frac{\partial }{\partial x}(\rho u^{3}+3pu)-2uF\right]\right\}=0; \end{array}$$

(energy equation)

3.9 $$\begin{array}{rl} & \frac{\partial }{\partial t}\left\{\rho u^{2}+3p-\tau \left[\frac{\partial }{\partial t}(\rho u^{2}+3p)+\frac{\partial }{\partial x}(\rho u^{3}+5pu)-2Fu\right]\right\}\\ & \;+\frac{\partial }{\partial x}\{\rho u^{3}+5pu-\tau [\frac{\partial }{\partial t}(\rho u^{3}+5pu)+\frac{\partial }{\partial x}\left(\rho u^{4}+8pu^{2}+5\frac{p^{2}}{\rho }\right)\\ & \;-F\left(3u^{2}+5\frac{p}{\rho }\right)]\}-2uF+2\tau \frac{F}{\rho }\left[\frac{\partial }{\partial t}(\rho u)+\frac{\partial }{\partial x}(\rho u^{2}+p)-\rho F\right]=0. \end{array}$$

Then introduce the coordinate system moving along the positive *χ*-direction of the one-dimensional space with the velocity *C*=*u*_0_ which is equal to phase velocity of the investigated quantum object

3.10 ξ=x−Ct.

Taking into account the de Broglie relation, we write that the group velocity *u*_*g*_ should be equal 2*u*_0_. Really, let us write down the energy of the relativistic particle

3.11 $$E=mc^{2},$$

where

3.12 $$m=\frac{m_{0}}{\sqrt{1-(v_{g}^{2}/c^{2})}},$$

where *c* is the light velocity, *v*_*g*_ is the group velocity and *m*_0_ is the mass of the rest for particle under study. Rewrite (3.11) as follows:

3.13 $$E=p\frac{c^{2}}{v_{g}},$$

where

3.14 $$p=mv_{g}$$

is the particle impulse. In the non-relativistic approximation, we have from (3.13)

3.15 $$E=\frac{1}{2}m_{0}v_{g}^{2}.$$

Using the principle of wave-particle parallelism in the de Broglie interpretation, we have for the energy of a particle

3.16 $$E=\hbar \omega =\hbar kv_{\phi },$$

where *ω* is the angular frequency, *v*_*ϕ*_=*ω*/*κ* is the phase velocity, *κ*=2*π*/λ is the wavenumber and λ is the wavelength. Correspondingly, the particle impulse *p* is

3.17 p=ℏk,

and using (3.17), we find

3.18 $$E=pv_{\phi }.$$

Then in the non-relativistic approach

3.19 $$E=\frac{1}{2}m_{0}v_{g}^{2}=\frac{1}{2}pv_{g}.$$

Compare (3.18) и (3.19), we find in the non-relativistic approximation

3.20 $$v_{\phi }=\frac{1}{2}v_{g}.$$

Then in the coordinate system moving with the phase velocity, indestructible soliton has the velocity which is equal to the phase velocity.

We extend the usual definition of the soliton object, which should satisfy two important conditions.

(1) In a moving coordinate system, this object is located in the same restricted area for all time moments including the movement under the influence of external forces.
(2) In the coordinate system moving with the phase velocity, indestructible soliton has the velocity which is equal to the phase velocity for all parts of a moving object.

Therefore, we use the moving coordinate system *ξ*=*x*−*ut*. In this system, all dependent hydrodynamic values are functions of (*ξ*,*t*). We investigate the possibility of the soliton creation. For this type of solution, the explicit time dependence does not exist in the considered coordinate system. As a result, equations (3.7)–(3.9) can be written as

(continuity equation)

3.21 $$\rho \frac{\partial u}{\partial \xi }-\tau \rho {\left(\frac{\partial u}{\partial \xi }\right)}^{2}-\frac{\partial }{\partial \xi }\left\{\tau \left[\frac{\partial p}{\partial \xi }-F\right]\right\}=0,$$

(motion equation)

3.22 $$\begin{array}{rl} & u\frac{\partial }{\partial \xi }\left\{\tau \left[\frac{\partial p}{\partial \xi }-F\right]\right\}-F\left[1-\tau \frac{\partial u}{\partial \xi }\right]+\rho u\frac{\partial u}{\partial \xi }+\frac{\partial p}{\partial \xi }-\tau \rho u{\left(\frac{\partial u}{\partial \xi }\right)}^{2}-\frac{\partial }{\partial \xi }\left\{\tau \left[3p\frac{\partial u}{\partial \xi }+2u\left(\frac{\partial p}{\partial \xi }-F\right)\right]\right\}=0, \end{array}$$

(energy equation)

3.23 $$\begin{array}{rl} & \rho u^{2}\frac{\partial u}{\partial \xi }+2u\frac{\partial p}{\partial \xi }+5p\frac{\partial u}{\partial \xi }-2Fu-\tau \rho u^{2}{\left(\frac{\partial u}{\partial \xi }\right)}^{2}-u\frac{\partial }{\partial \xi }\left\{\tau u\frac{\partial p}{\partial \xi }\right\}-3\tau u\frac{\partial p}{\partial \xi }\frac{\partial u}{\partial \xi }-6u\frac{\partial }{\partial \xi }\left\{\tau p\frac{\partial u}{\partial \xi }\right\}\\ & \;-11\tau p{\left(\frac{\partial u}{\partial \xi }\right)}^{2}-5\frac{\partial }{\partial \xi }\left\{\tau \frac{\partial }{\partial \xi }\left(\frac{p^{2}}{\rho }\right)\right\}+u\frac{\partial }{\partial \xi }\{\tau Fu\}+5\tau Fu\frac{\partial u}{\partial \xi }+5\frac{\partial }{\partial \xi }\left\{\tau F\frac{p}{\rho }\right\}+2\tau \frac{F}{\rho }\left(\frac{\partial p}{\partial \xi }-F\right)=0. \end{array}$$

Write down the system of equations (3.21)–(3.23) in the dimensionless form. All dimensionless values are marked by a tilde. Introduce the scales *ρ*→*ρ*_0_, *u*→*u*_0_, $p\to \rho_{0}u_{0}^{2}$, *ξ*→*ξ*_0_. We use the approximation of the non-locality parameter *τ* in the form

3.24 $$\tau =\frac{\hbar }{mu^{2}},$$

where ℏ is the Plank constant.

The dimensionless continuity equation is rewritten as

3.25 $$\begin{array}{r} \tilde{\rho }\frac{\partial \tilde{u}}{\partial \tilde{\xi }}-\frac{\hbar }{m{\tilde{u}}^{2}}\frac{1}{u_{0}\xi_{0}}\tilde{\rho }{\left(\frac{\partial \tilde{u}}{\partial \tilde{\xi }}\right)}^{2}-\frac{\hbar }{mu_{0}}\frac{1}{\xi_{0}}\frac{\partial }{\partial \tilde{\xi }}\left\{\frac{1}{{\tilde{u}}^{2}}\left[\frac{\partial \tilde{p}}{\partial \tilde{\xi }}-\frac{\xi_{0}}{\rho_{0}u_{0}^{2}}F\right]\right\}=0. \end{array}$$

Introduce the parameter

3.26 $$H=\frac{\hbar }{mu_{0}\xi_{0}}$$

and (3.25) and (3.26) yield

3.27 $$\tilde{\rho }\frac{\partial \tilde{u}}{\partial \tilde{\xi }}-H\frac{1}{{\tilde{u}}^{2}}\tilde{\rho }{\left(\frac{\partial \tilde{u}}{\partial \tilde{\xi }}\right)}^{2}-H\frac{\partial }{\partial \tilde{\xi }}\left\{\frac{1}{{\tilde{u}}^{2}}\left[\frac{\partial \tilde{p}}{\partial \tilde{\xi }}-\tilde{F}\right]\right\}=0.$$

The force scale *F*_0_ is used in (3.27)

3.28 $$F_{0}=\frac{\xi_{0}}{\rho_{0}u_{0}^{2}}.$$

The motion equation

3.29 $$\begin{array}{rl} & H\tilde{u}\frac{\partial }{\partial \tilde{\xi }}\left\{\frac{1}{{\tilde{u}}^{2}}\left[\frac{\partial \tilde{p}}{\partial \tilde{\xi }}-\tilde{F}\right]\right\}-\tilde{F}\left[1-H\frac{1}{{\tilde{u}}^{2}}\frac{\partial \tilde{u}}{\partial \tilde{\xi }}\right]+\tilde{\rho }\tilde{u}\frac{\partial \tilde{u}}{\partial \tilde{\xi }}+\frac{\partial \tilde{p}}{\partial \tilde{\xi }}-H\frac{1}{\tilde{u}}\tilde{\rho }{\left(\frac{\partial \tilde{u}}{\partial \tilde{\xi }}\right)}^{2}\\ & \;-H\frac{\partial }{\partial \tilde{\xi }}\left\{\frac{1}{{\tilde{u}}^{2}}\left[3\tilde{p}\frac{\partial \tilde{u}}{\partial \tilde{\xi }}+2\tilde{u}\left(\frac{\partial \tilde{p}}{\partial \tilde{\xi }}-\tilde{F}\right)\right]\right\}=0, \end{array}$$

and the energy equation

3.30 $$\begin{array}{rl} & \tilde{\rho }{\tilde{u}}^{2}\frac{\partial \tilde{u}}{\partial \tilde{\xi }}+2\tilde{u}\frac{\partial \tilde{p}}{\partial \tilde{\xi }}+5\tilde{p}\frac{\partial \tilde{u}}{\partial \tilde{\xi }}-2\tilde{F}\tilde{u}-H\tilde{u}\frac{\partial }{\partial \xi }\left\{\frac{1}{\tilde{u}}\frac{\partial \tilde{p}}{\partial \tilde{\xi }}\right\}-6H\tilde{u}\frac{\partial }{\partial \tilde{\xi }}\left\{\frac{1}{{\tilde{u}}^{2}}\tilde{p}\frac{\partial \tilde{u}}{\partial \tilde{\xi }}\right\}-3H\frac{1}{\tilde{u}}\frac{\partial \tilde{p}}{\partial \tilde{\xi }}\frac{\partial \tilde{u}}{\partial \tilde{\xi }}\\ & \;-11H\frac{1}{{\tilde{u}}^{2}}\tilde{p}{\left(\frac{\partial \tilde{u}}{\partial \tilde{\xi }}\right)}^{2}-H\tilde{\rho }{\left(\frac{\partial \tilde{u}}{\partial \tilde{\xi }}\right)}^{2}-5H\frac{\partial }{\partial \tilde{\xi }}\left\{\frac{1}{{\tilde{u}}^{2}}\frac{\partial }{\partial \tilde{\xi }}\left(\frac{{\tilde{p}}^{2}}{\tilde{\rho }}\right)\right\}+H\tilde{u}\frac{\partial }{\partial \tilde{\xi }}\left\{\frac{1}{\tilde{u}}\tilde{F}\right\}+5H\frac{1}{\tilde{u}}\tilde{F}\frac{\partial \tilde{u}}{\partial \tilde{\xi }}\\ & \;+5H\frac{\partial }{\partial \tilde{\xi }}\left\{\frac{1}{{\tilde{u}}^{2}}\tilde{F}\frac{\tilde{p}}{\tilde{\rho }}\right\}+2H\frac{1}{{\tilde{u}}^{2}}\frac{\tilde{F}}{\tilde{\rho }}\left(\frac{\partial \tilde{p}}{\partial \tilde{\xi }}-\tilde{F}\right)=0, \end{array}$$

are subjected to the same transformations.

In the Rayleigh theory, the value $\tilde{F}$ is the constant dimensionless parameter. The solution of equations (3.27), (3.29) and (3.30) can be simplified after transformation of the mentioned equations into the one parametric system using the special choice of the lengthscale *ξ*_0_. Namely

3.31 $$\xi_{0}=\frac{\hbar }{mu_{0}},$$

then *H*=1, and the force scale is

3.32 $$F_{0}=\rho_{0}u_{0}^{3}\frac{m}{\hbar }\left[\frac{\text{dyne}}{{\text{cm}}^{3}}\right].$$

The introduced scales have the transparent physical sense. Really, let us introduce the quantum Reynolds number and transform this number using the introduced scales:

3.33 $$Re=\frac{\rho_{0}u_{0}\xi_{0}}{\mu_{0}}=\frac{\rho_{0}u_{0}\xi_{0}}{\upsilon_{0}\rho_{0}}=\frac{u_{0}\xi_{0}}{\upsilon_{0}}=\frac{u_{0}}{\upsilon_{0}}\frac{\hbar }{mu_{0}}=\frac{mu_{0}}{\hbar }\frac{\hbar }{mu_{0}}=1.$$

Then we are dealing with the matter flow for *Re*=1.

We need to solve the following system of equations:

3.34 $$\begin{array}{rl} & \tilde{\rho }\frac{\partial \tilde{u}}{\partial \tilde{\xi }}-\frac{1}{{\tilde{u}}^{2}}\tilde{\rho }{\left(\frac{\partial \tilde{u}}{\partial \tilde{\xi }}\right)}^{2}-\frac{\partial }{\partial \tilde{\xi }}\left\{\frac{1}{{\tilde{u}}^{2}}\left[\frac{\partial \tilde{p}}{\partial \tilde{\xi }}-\tilde{F}\right]\right\}=0, \end{array}$$

3.35 $$\begin{array}{rl} & \tilde{u}\frac{\partial }{\partial \tilde{\xi }}\left\{\frac{1}{{\tilde{u}}^{2}}\left[\frac{\partial \tilde{p}}{\partial \tilde{\xi }}-\tilde{F}\right]\right\}-\tilde{F}\left[1-\frac{1}{{\tilde{u}}^{2}}\frac{\partial \tilde{u}}{\partial \tilde{\xi }}\right]+\tilde{\rho }\tilde{u}\frac{\partial \tilde{u}}{\partial \tilde{\xi }}+\frac{\partial \tilde{p}}{\partial \tilde{\xi }}\\ & \;-\frac{1}{\tilde{u}}\tilde{\rho }{\left(\frac{\partial \tilde{u}}{\partial \tilde{\xi }}\right)}^{2}-\frac{\partial }{\partial \tilde{\xi }}\left\{\frac{1}{{\tilde{u}}^{2}}\left[3\tilde{p}\frac{\partial \tilde{u}}{\partial \tilde{\xi }}+2\tilde{u}\left(\frac{\partial \tilde{p}}{\partial \tilde{\xi }}-\tilde{F}\right)\right]\right\}=0 \end{array}$$

3.36 $$\begin{array}{rl} \text{and}\; & \tilde{\rho }{\tilde{u}}^{2}\frac{\partial \tilde{u}}{\partial \tilde{\xi }}+2\tilde{u}\frac{\partial \tilde{p}}{\partial \tilde{\xi }}+5\tilde{p}\frac{\partial \tilde{u}}{\partial \tilde{\xi }}-2\tilde{F}\tilde{u}-\tilde{u}\frac{\partial }{\partial \xi }\left\{\frac{1}{\tilde{u}}\frac{\partial \tilde{p}}{\partial \tilde{\xi }}\right\}-6\tilde{u}\frac{\partial }{\partial \tilde{\xi }}\left\{\frac{1}{{\tilde{u}}^{2}}\tilde{p}\frac{\partial \tilde{u}}{\partial \tilde{\xi }}\right\}-3\frac{1}{\tilde{u}}\frac{\partial \tilde{p}}{\partial \tilde{\xi }}\frac{\partial \tilde{u}}{\partial \tilde{\xi }}-11\frac{1}{{\tilde{u}}^{2}}\tilde{p}{\left(\frac{\partial \tilde{u}}{\partial \tilde{\xi }}\right)}^{2}\\ & \;-\tilde{\rho }{\left(\frac{\partial \tilde{u}}{\partial \tilde{\xi }}\right)}^{2}-5\frac{\partial }{\partial \tilde{\xi }}\left\{\frac{1}{{\tilde{u}}^{2}}\frac{\partial }{\partial \tilde{\xi }}\left(\frac{{\tilde{p}}^{2}}{\tilde{\rho }}\right)\right\}+\tilde{u}\frac{\partial }{\partial \tilde{\xi }}\left\{\frac{1}{\tilde{u}}\tilde{F}\right\}+5\frac{1}{\tilde{u}}\tilde{F}\frac{\partial \tilde{u}}{\partial \tilde{\xi }}\\ & \;+5\frac{\partial }{\partial \tilde{\xi }}\left\{\frac{1}{{\tilde{u}}^{2}}\tilde{F}\frac{\tilde{p}}{\tilde{\rho }}\right\}+2\frac{1}{{\tilde{u}}^{2}}\frac{\tilde{F}}{\tilde{\rho }}\left(\frac{\partial \tilde{p}}{\partial \tilde{\xi }}-\tilde{F}\right)=0. \end{array}$$

## 4. Numerical simulation

Equations (3.34)–(3.36) constitute the one parametric Cauchy problem as the system of the three ordinary differential equations of the second order. Technical computing software Maple allows the realization of the vast mathematical modelling using the variation of the six Cauchy conditions and the $\tilde{F}$ parameter. Let us show the results of some calculations using the Maple notations: $\tilde{\xi }\to t$, $\tilde{u}(\tilde{\xi })\to \text{u(t)}$, $\tilde{\rho }(\tilde{\xi })\to \text{r(t)}$, $\tilde{p}(\tilde{\xi })\to \text{p(t)}$ and $\tilde{F}\to \text{F}$. For the chosen Cauchy conditions, we find

4.1 r(0)=1,u(0)=1,p(0)=1,D(r)(0)=0,D(u)(0)=0,D(p)(0)=0.

Figures 1–10 contain the result of calculations for the following set of $\tilde{F}$ data, namely $\tilde{F}$ is equal to: 0.001; 0.01; 0.1; 0; 1; 10; 100; 1000; 10^4^, 10^5^.

**Figure 1.**
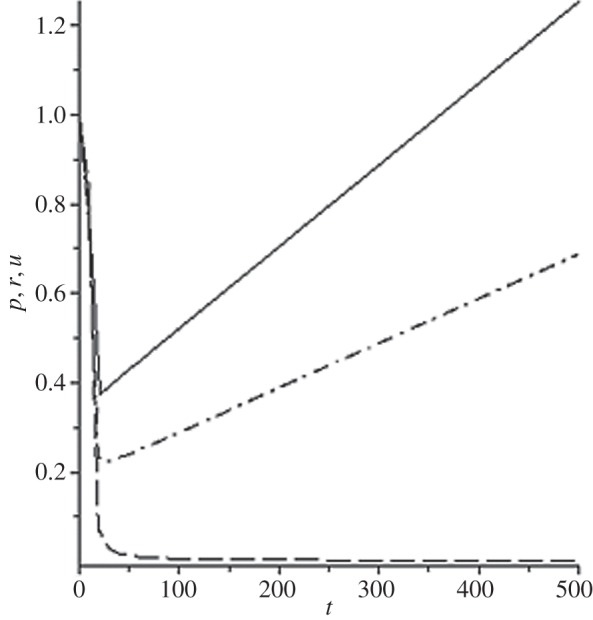
The change of the density (solid line), the pressure (dashed-dotted line), and the velocity (dashed line) in the moving coordinate system at $\tilde{F}=0.001$.

**Figure 2.**
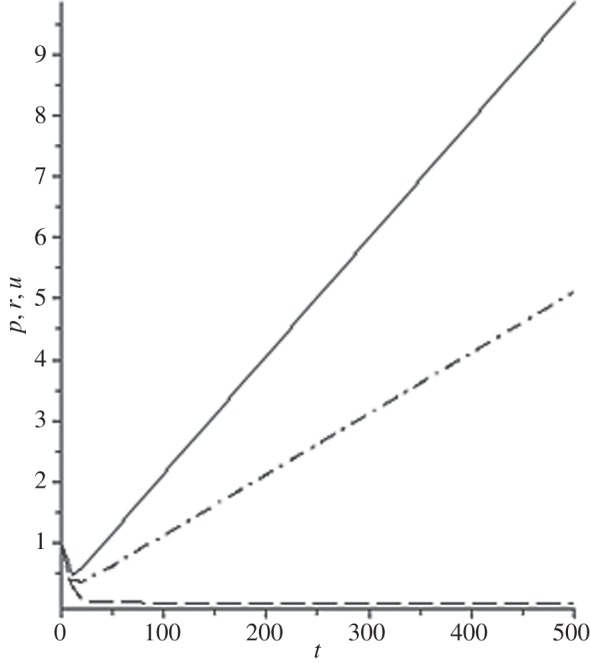
The change of the density (solid line), the pressure (dashed-dotted line), and the velocity (dashed line) in the moving coordinate system at $\tilde{F}=0.01$.

**Figure 3.**
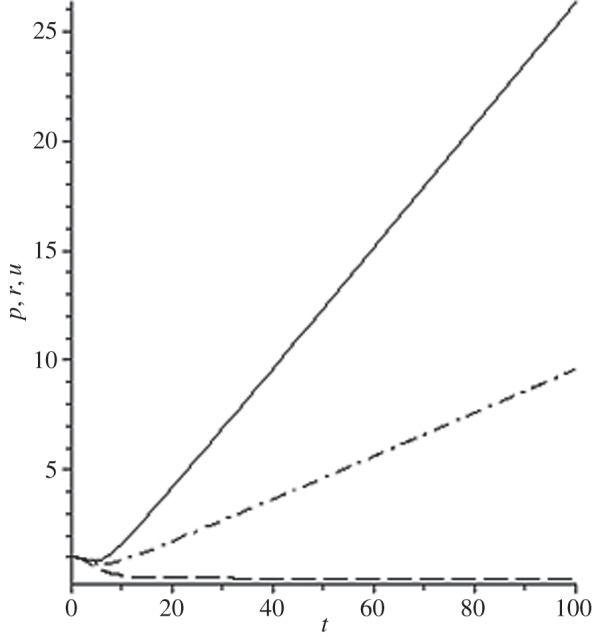
The change of the density (solid line), the pressure (dashed-dotted line), and the velocity (dashed line) in the moving coordinate system at $\tilde{F}=0.1$.

**Figure 4.**
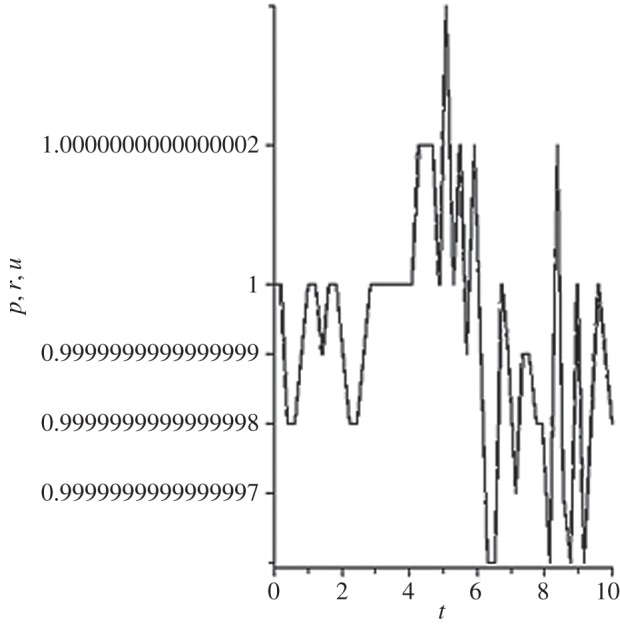
The change of the density (solid line), the pressure (dashed-dotted line), and the velocity (dashed line) in the moving coordinate system at $\tilde{F}=0$.

**Figure 5.**
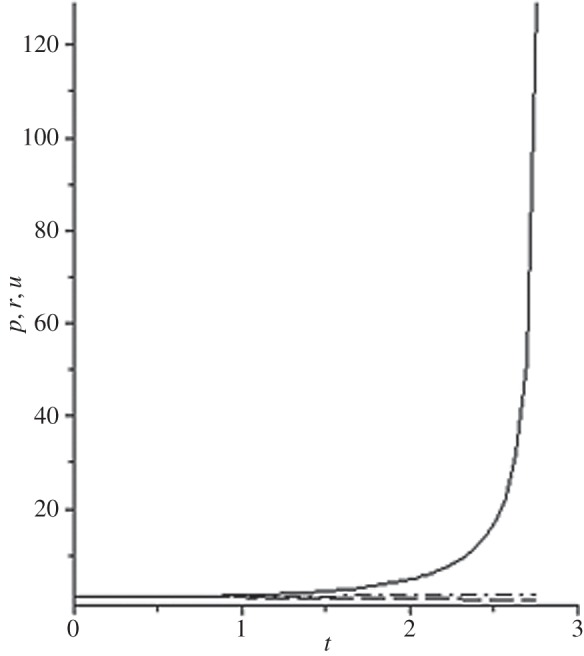
The change of the density (solid line), the pressure (dashed-dotted line), and the velocity (dashed line) in the moving coordinate system at $\tilde{F}=1$.

**Figure 6.**
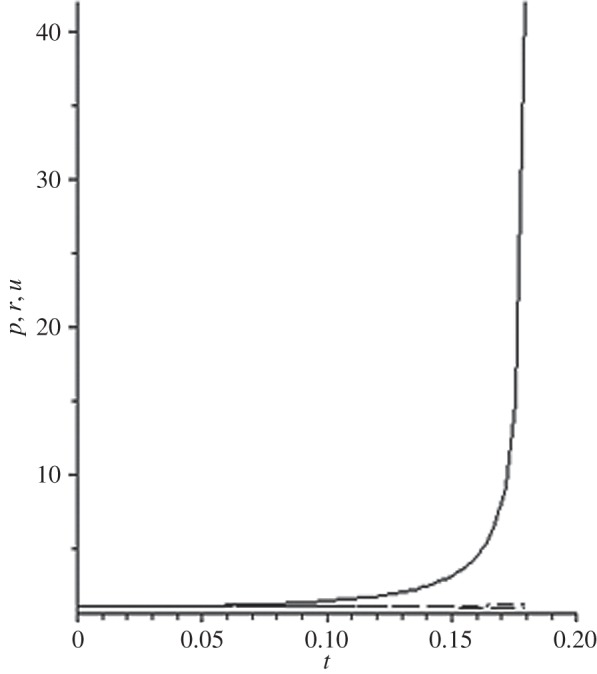
The change of the density (solid line), the pressure (dashed-dotted line), and the velocity (dashed line) in the moving coordinate system at $\tilde{F}=10$.

**Figure 7.**
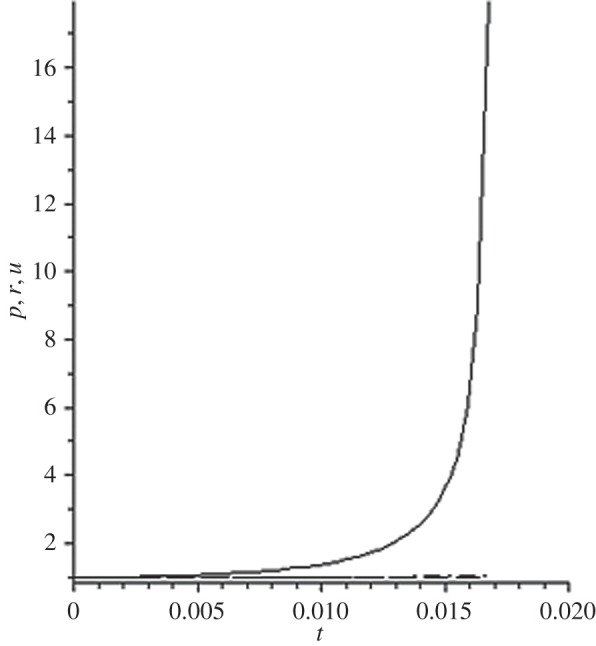
The change of the density (solid line), the pressure (dashed-dotted line), and the velocity (dashed line) in the moving coordinate system at $\tilde{F}=100$.

**Figure 8.**
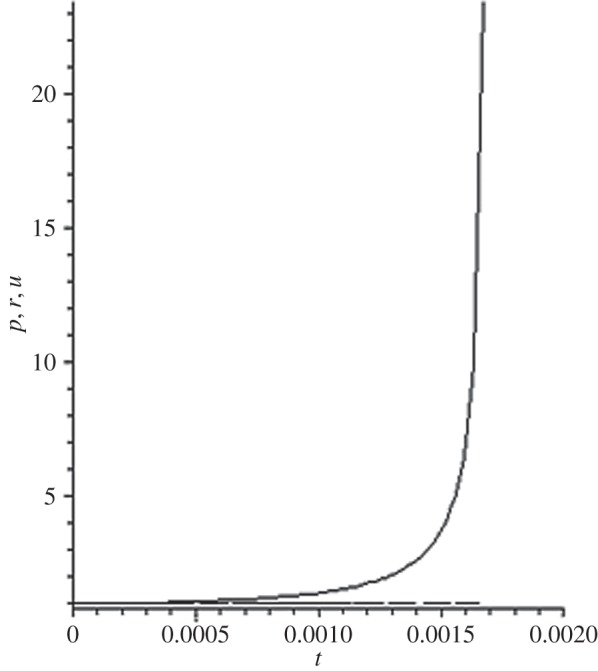
The change of the density (solid line), the pressure (dashed-dotted line), and the velocity (dashed line) in the moving coordinate system at $\tilde{F}=1000$.

**Figure 9.**
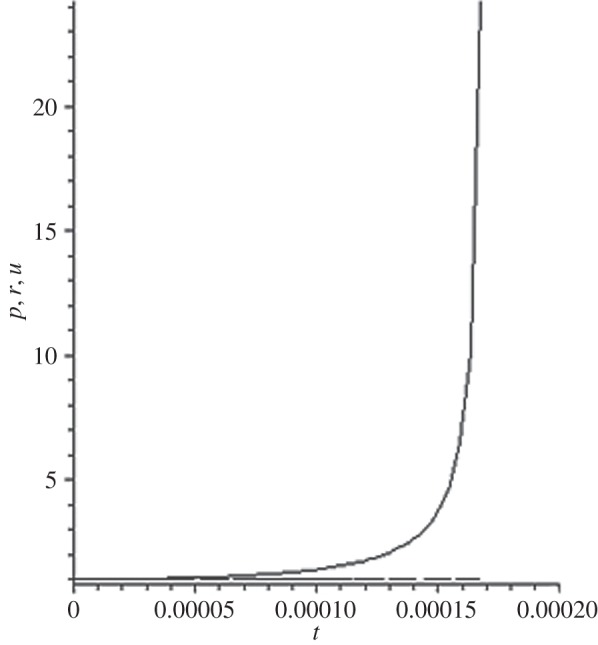
The change of the density (solid line), the pressure (dashed-dotted line), and the velocity (dashed line) in the moving coordinate system at $\tilde{F}=10\;000$.

**Figure 10.**
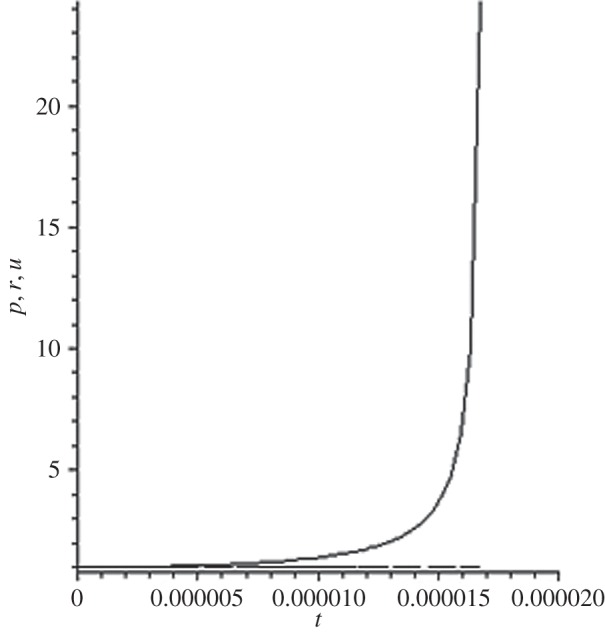
The change of the density (solid line), the pressure (dashed-dotted line), and the velocity (dashed line) in the moving coordinate system at $\tilde{F}=10^{5}$.

## 5. Discussion

Parameter $\tilde{F}$ defines the force of the sound action on matter; varying over eight orders of this parameter $\tilde{F}$ leads to the radical reconstruction of the flow. Namely,

(1) at the condition $\tilde{F}<1$, there are no solitons (figures 1–3);
(2) the condition $\tilde{F}=0$ leads to the trivial solution $\tilde{u}=\tilde{\rho }=\tilde{p}=1$ (figure 4). Figure 4 is given for the demonstration of the high accuracy of the numerical method (variant of the Runge–Kutta method); and
(3) the condition $\tilde{F}>1$ leads to the soliton creation (figures 5–10).

The structure of the creating solitons has the following very remarkable features.

(1) As it can be expected in the soliton theory, all soliton parts move with the same velocity—the condition $\tilde{u}=1$ fulfils with high accuracy. The soliton is placed in the bounded region of space. It is important to underline that we deal with the Cauchy problem. It means that the mentioned effect is a product of the matter of self-organization.
(2) The linear soliton size diminishes with the $\tilde{F}$ increase. Let us give the concrete data:
  if $\tilde{F}=1\to {\tilde{\xi }}_{\lim}=2.797$; if $\tilde{F}=10\to {\tilde{\xi }}_{\lim}=0.182$; if $\tilde{F}=100\to {\tilde{\xi }}_{\lim}=0.017131754$;
  if $\tilde{F}=1000\to {\tilde{\xi }}_{\lim}=0.0017028$; if $\tilde{F}=10\;000\to {\tilde{\xi }}_{\lim}=0.00017017951$; if $\tilde{F}=10^{5}\to {\tilde{\xi }}_{\lim}=0.000017\;016920$.
(3) If $\tilde{F}>100$, then $\tilde{F}$ increasing 10-fold leads to diminishing 10-fold less of the soliton size. It means that for the large $\tilde{F}$, the value $\tilde{x}$ is close to $\tilde{t}$.
(4) We can watch the gross density change on the soliton front without significant pressure changing. It is the desired effect which the CF adherents try to prove. Let us deliver the concrete numerical results because the graphic illustrations (see also figures 5–10) do not reflect the scale of dramatic change.
  For $\tilde{F}=100$ and $\tilde{\xi }=0.00171317539$, we find $\tilde{p}=1.000884428$, $\tilde{u}=0.990726$ and $\tilde{\rho }=2.7810799\times 10^{7}$. For the point placed closer to ${\tilde{\xi }}_{\lim}=0.017131754$, we have: if $\tilde{\xi }=0.0017131753995$, then $\tilde{p}=1.000884429$, $\tilde{u}=0.990726$ and $\tilde{\rho }=4.4694082\times 10^{7}$.
  For $\tilde{F}=1000$ and $\tilde{\xi }=0.001702$, we find $\tilde{p}=1.000887$, $\tilde{u}=0.999907$ and $\tilde{\rho }=819.0197$.
  For $\tilde{F}=10\;000$ and $\tilde{\xi }=0.0001701795$, we have $\tilde{p}=1.0000889$, $\tilde{u}=0.99990728$ and $\tilde{\rho }=3.63483052\times 10^{6}$.
  For $\tilde{F}=10^{5}$ (${\tilde{\xi }}_{\lim}=0.17016920\times 10^{-4})$ and $\tilde{\xi }=0.17016919\times 10^{-4}$, we find $\tilde{p}=1.0000088907$, $\tilde{u}=0.9999907284$ and $\tilde{\rho }=1.2247653\times 10^{7}$. If $\tilde{\xi }=0.1701691955\times 10^{-4}$, then $\tilde{p}=1.0000088907$, $\tilde{u}=0.9999907284$ and $\tilde{\rho }=5.05040680\times 10^{8}$.
(5) The density increase in the milliard times on the soliton front can lead to the effect of CF. If required, the density can be normalized to the total mass $\tilde{M}$ (known from an experiment) which falls on the unit of the wavefront area:
  5.1 $$\tilde{M}=\int_{0}^{\xi_{\exp}}\tilde{\rho }\text{d}\tilde{\xi }.$$

Let us go now to the formal solution of so-called ‘classical’ hydrodynamic equations in the frame of choosing of the plane travelling longitudinal wave. It seems that for this case, we should solve numerically the system of equations (3.27), (3.29) and (3.30) for *H*=0. But we need not to do it because the analytical solution can be found. Really, the system of equation has the form for the mentioned case:

(continuity equation)

5.2 $$-u\frac{\partial \rho }{\partial \xi }+\frac{\partial }{\partial \xi }(\rho u)=0,$$

(motion equation)

5.3 $$-u\frac{\partial }{\partial \xi }(\rho u)-F+\frac{\partial }{\partial \xi }(\rho u^{2}+p)=0,$$

(energy equation)

5.4 $$-u\frac{\partial }{\partial \xi }(\rho u^{2}+3p)+\frac{\partial }{\partial \xi }(\rho u^{3}+5pu)-2Fu=0.$$

Equation (5.2) yields

5.5 $$\rho \frac{\partial u}{\partial \xi }=0$$

or

5.6 u=const.

From the motion equation (5.3) follows

5.7 $$\frac{\partial p}{\partial \xi }=F.$$

Energy equation (5.4) does not deliver a new independent relation and returns us to the relation (5.7). Then for the constant *F*, equation (5.7) leads to the trivial (and as we see, wrong) dependence, which does not contain the matter density:

5.8 p=Fξ+const.

## 6. Conclusion

In spite of all experimental problems and difficulties, the cavitation-induced fusion or generally speaking, acoustic cold fusion (ACF) has serious experimental confirmation. There is the obvious contradiction between the mentioned experimental results and conclusions of the classical local hydrodynamics. As we see, local hydrodynamics is not applicable to the description of the ACF in principal. The realized mathematical modelling leads to the gross density change on the soliton front without significant pressure changing. It is the desired effect which the CF adherents try to prove. Then the following step of the investigation consists in the numerical solution of the three-dimensional non-stationary boundary problem on the basement of unified non-local transport theory.
